# Poor livestock keepers: ecosystem–poverty–health interactions

**DOI:** 10.1098/rstb.2016.0166

**Published:** 2017-06-05

**Authors:** Delia Grace, Johanna Lindahl, Francis Wanyoike, Bernard Bett, Tom Randolph, Karl M. Rich

**Affiliations:** 1 International Livestock Research Institute, Box 30709, Nairobi, Kenya; 2 Department of Medical Biochemistry and Microbiology, Uppsala University, 751 05 Uppsala, Sweden; 3 Lincoln University, Lincoln 7647, New Zealand

**Keywords:** poverty, livestock keepers, zoonoses, ecosystems, system dynamics

## Abstract

Humans have never been healthier, wealthier or more numerous. Yet, present success may be at the cost of future prosperity and in some places, especially in sub-Saharan Africa, poverty persists. Livestock keepers, especially pastoralists, are over-represented among the poor. Poverty has been mainly attributed to a lack of access, whether to goods, education or enabling institutions. More recent insights suggest ecosystems may influence poverty and the self-reinforcing mechanisms that constitute poverty traps in more subtle ways. The plausibility of zoonoses as poverty traps is strengthened by landmark studies on disease burden in recent years. While in theory, endemic zoonoses are best controlled in the animal host, in practice, communities are often left to manage disease themselves, with the focus on treatment rather than prevention. We illustrate this with results from a survey on health costs in a pastoral ecosystem. Epidemic zoonoses are more likely to elicit official responses, but these can have unintended consequences that deepen poverty traps. In this context, a systems understanding of disease control can lead to more effective and pro-poor disease management. We illustrate this with an example of how a system dynamics model can help optimize responses to Rift Valley fever outbreaks in Kenya by giving decision makers real-time access to the costs of the delay in vaccinating. In conclusion, a broader, more ecological understanding of poverty and of the appropriate responses to the diseases of poverty can contribute to improved livelihoods for livestock keepers in Africa.

This article is part of the themed issue ‘One Health for a changing world: zoonoses, ecosystems and human well-being’.

## 1. Introduction: an overall healthier, wealthier world

In a world that is ever wealthier, why do so many livestock keepers remain poor? This paper examines poverty among livestock keepers with a focus on pastoralists in Africa, a group of livestock keepers who rank among the richest in terms of animals kept per household but among the worst-off in terms of human development. In this paper, we briefly consider conventional and emerging explanations for persistent poverty and then muster the evidence that, in livestock-keeping communities, diseases may have a unique role in trapping people in poverty, and that these traps may be hardest to escape where ecosystems are most stressed or disturbed.

From a perspective of centuries and millennia, humans have never been healthier, wealthier or more numerous. The world population reached 7.3 billion in mid-2015 and is predicted to reach 8.5 billion in 2030 and 11.2 billion by 2100 [1]. Population growth will be fastest in Africa, predicted to be home to 4 billion people by 2100: a dramatic increase from around 100 million in 1800. Yet, despite unprecedented increases in population, the proportion of the world living in extreme poverty is declining: in 2015, for the first time in history, less than one in 10 lived in absolute poverty [2]. Other development indicators are likewise encouraging: in 2015, 91% of the global population used an improved drinking water source, with 2.6 billion people gaining access since 1990 [3], while the number of hungry people had dropped to 795 million [4], much less than the more than 2.4 billion who were overweight or obese [5]. Also in 2015, primary education enrolment reached 91% in developing countries, while gender gaps continued to decline and long-term trends driving the waning of war remained strong [6,7].

In parallel to other improvements in human development, the last few centuries have seen dramatic increases in longevity and declines in communicable illnesses. Life expectancy increased by 5 years between 2000 and 2015, the fastest increase since the 1960s [8]: for the first time, the average child born in 2015 can expect to live for 70 years. While disease from all causes is trending down, the communicable and nutritional diseases associated with poverty are decreasing relative to non-communicable diseases such as diabetes and cardiovascular disease, which are more likely to be associated with poor life choices than deprivation [9,10].

Will human development continue to relentlessly improve or do we risk exceeding Earth's carrying capacity? [11,12]. On the one hand, history is littered with fears of disastrous anthropogenic change that did not come to pass: from trains that did not cause milk to turn bad and passengers to go blind, to cities that did not drown in horse manure; and from ice ages that did not return to scarce mineral resources that remain unexhausted. On the other hand, things that cannot go on forever stop: often in abrupt and unpleasant ways. Four critical environmental thresholds may have already been breached, namely: climate change, loss of biosphere integrity, land-system change and altered phosphorus and nitrogen cycles [12]. Other existential threats include civilization-altering plagues [13] and a reversal of the last epidemiological transition as the result of widespread pathogen resistance to antimicrobial drugs [14].

Against a background of uncertainty over the sustainability of human development, the rest of this paper discusses poverty in the context of livestock keeping, and the ecosystem and health interactions that may trap people in poverty or help them to escape. We use examples from a recently completed project in Africa (Dynamic Disease Drivers in Africa Consortium (DDDAC)) to explore how One Health and systems understanding can broaden our understanding of how disease, and responses to disease, affect poor livestock keepers. The DDDAC project investigated the link between ecosystem disturbance, impairment of ecosystem services and health and well-being outcomes; in the Kenyan case study featured in this paper, the ecosystem change was the introduction of irrigation to arid, pastoral lands.

## 2. Poor livestock keepers

Livestock production constitutes around 40% of global agricultural gross domestic product, but households dependent on livestock, especially small-scale livestock keepers and pastoralists, are over-represented among poor households. Increasingly, poverty is concentrated in sub-Saharan Africa, where population growth exceeds the rate of poverty reduction, while education, healthcare, housing and technology use continue to lag behind other developing regions [15] and poverty among livestock keepers is also deepest in this continent.

Small-scale livestock keepers include agro-pastoralists, rural landless and the peri-urban poor who keep a few livestock as part of a diverse livelihood strategy. They are found in all countries, but are most heavily concentrated in Asia and Africa: estimates suggest from 750 million to over 1 billion people are in these households [16]. Small-scale livestock keeping has been pessimistically portrayed as a symptom of poverty and optimistically as a pathway out of it [17]. Likewise, small-scale livestock keepers are variously seen as custodians of sustainable agro-ecosystems or the combined victims and perpetrators of unsustainable agriculture.

Smallholder farms usually integrate crops and livestock, so they can harness ecological processes such as nutrient recycling and use of crop by-products. Food waste is low as livestock products are mainly destined to local markets and poor consumers. Small-scale farming creates habit heterogeneity and semi-natural environments that benefit biodiversity and ecosystem services that rely on biodiversity. Because small farms are less coupled to financial and commodity markets they are less vulnerable to the price volatility that characterizes much livestock production, and the embedding of smallholder production in centuries old rural tradition provides social and cultural stability. However, small scale is often associated with weak financial viability, productivity is much lower than intensive farms, yield gaps are high and small farmers face difficulty in meeting the sanitary measures and regulations demanded in long-chain markets [18].

Pastoralism is mostly found in the developing world, in areas where intensive crop cultivation is limited or physically not possible and estimates of people involved in pastoralism vary from 50 to 200 million [19,20]. Pastoral systems are found in the arid zones with low and irregular rainfall, water and natural forage resources. In these areas, they are one of the main economic activities on which the poorest populations are dependent as a source of food and cash income. Pastoral systems have low levels of productivity in physical terms due to their dependence on often poor quality and scarce local resources and limited access to purchased inputs, resulting in both low levels of overall inputs used and output produced. They are often characterized by high poverty, chronic conflict and low governance. However, pastoralists have proven remarkably resilient in surviving and even thriving in some of the most challenging terrestrial ecosystems. Moreover, pastoralism can be modern, efficient and highly profitable and out-compete the alternatives many times over [21].

## 3. The poverty puzzle: assets, institutions and ecosystems

Livestock keepers, especially pastoralists, are over-represented among the poor and the poorest of the poor. General explanations for poverty have often focused on deficiencies or lacks: especially, a lack of assets and a lack of knowledge and skills. These perceived lacks naturally led to solutions premised on providing: whether infrastructure and material goods, or education, training and capacity-building. Commonly, the things to be provided are based on models prevalent in the western world, which first broke free of pervasive poverty. Yet, provision has not proven a reliable way of remediating persistent poverty in livestock-dependent agro-ecosystems.

This failure or imperfect success led to increased interest in the role of institutions in reducing poverty. Indeed, there are strong empirical relations between ‘good’ institutions (property rights, effective law enforcement, equity and efficient bureaucracies) and economic growth [22]. This finding led to interesting avenues for exploration: Where do institutions come from? And how can they be changed to help more people escape from poverty? Acemoglu *et al*. [23] argued that globalization patterns led to institutions that were conducive to entrenched poverty or the reverse. These patterns were in turn determined by geography, and, especially, by diseases present. For example, Australia had ecosystems suitable for European settlement and developed benign institutions. By contrast, Europeans in Nigeria faced high mortality rates, could not easily become settlers and so set up worse (extractive) institutions. Although superficially plausible, there are many confounding factors, and current institutional arrangements are not necessarily decisive in determining economic outcomes. For example, African ethnic groups, which extended across borders, had similar economic performance irrespective of the institutions of the country in which they ended up but reflecting their pre-colonial ethnic institutional traits [24]. Moreover, the results of attempting institutional change have been at best mixed: major institution-changing and -building initiatives in the African livestock sector (group ranches, cooperatives, veterinary privatization) have had little success.

The incompleteness of explanations based on deficits, whether of assets or institutions, is underlined not only by those who remain in poverty but by those who escape. Despite around 1 trillion USD of official aid, since 1970 [25] more than 40% of African people remain in severe poverty: more in absolute numbers than were poor in 1970. Meanwhile, in Asia, around a billion people have moved out of abject poverty, and progress has been greatest where material provision and institutional building by development actors has been least [26].

Deepening the discussion on institutions, culture and behaviour have also been hypothesized to play a role in poverty. This has sometimes been seen as ‘blaming the victim’ [27], but recently, sociologists and behavioural economists have returned to the possibility, finding empirical evidence that culture can influence poverty and its determinants; for example, influencing distribution of food aid among the Dinka [28]. The 2015 World Bank Development report [29] considers behavioural economics key to development and that understanding behavioural biases and heuristics can lead to more successful interventions. (The report also explores the biases of development professionals finding they often interpret data differently depending on the frame and have little idea about the opinions of the poor people they aim to help.) There is a rich literature on pastoralist societies and culture, and how failure to understand these has led to the failure of development projects as well as widespread misperceptions that pastoralism is backwards and environmentally damaging [21].

But where does culture come from? An intriguing recent paper links culture to agro-ecosystems: the authors found that people from rice-growing regions of China appear to think in more interdependent and holistic ways than those from wheat-growing areas, perhaps because it takes much more cooperation and overall effort to grow rice than wheat [30]. Rice-growing areas also have fewer patents, and fewer divorces, than wheat regions, which may also reflect lower innovation and higher conformity in rice cultures. Adding another twist, the behaviours that emerge in different agro-ecosystems, may, like most other behaviours, have genetic as well as environmental components. The so-called First Law of behaviour genetics states that all human traits are heritable [31]. A meta-analysis of 50 years of twin studies investigated the heritability of thousands of complex traits (*n* = 17 804). This found that across all traits (varying from cardiovascular to cognitive performance and from social values to weight maintenance), the reported heritability was 49% [32]. A recent study finds geography and ecology have more influenced the genetic make-up of human groups in southern Africa than languages or livelihood strategies [33]. Although not without controversy [34], an understanding of genetics is transforming our understanding of health and disease and might also mediate some relations between ecosystems and social outcomes [35,36].

## 4. Disease as a poverty trap

Another approach to understanding poverty that has received much attention in recent decades is especially relevant to livestock keepers. This is the hypothesis that poverty traps (self-reinforcing mechanisms through which poor individuals or countries remain poor) explain the persistence of poverty in an overall developing world. Self-reinforcing mechanisms imply threshold conditions under which the poor stay poor and over which the rich get richer. Poverty traps underpin ‘big push’ theories of development, such as the Millennium Villages. The issue is controversial, and empirical evidence mixed [37,38], but it seems poverty traps may be more important where households primarily rely on one asset such as livestock [39,40]. Different types of poverty traps have been described: economic, demographic, socio-political, behavioural, environmental and geographical. In this paper, we focus on infectious disease as a poverty trap. If disease is an important poverty trap, as argued by some, then controlling disease by itself may enable the poor to escape poverty traps [41,42], and one-time policy efforts to break the poverty trap may have lasting effects obviating the need for long-term provision of assets, capacity-building or institutions: an attractive proposition for development agents.

The basis for disease-driven poverty traps rests on three bodies of literature. Firstly, there is a strong association between extreme poverty, high prevalence of infectious diseases and ecological conditions suitable for pathogen development [43]. Pathogens can have a significant impact on nutrition and impair cognitive development, eroding the human capital that underpins development and escape from poverty. Moreover, the relation is bi-directional and poverty also increases exposure and susceptibility to pathogens. Secondly, the testimony of poor people, which has become increasingly emphasized in the development discourse and planning. Across dozens of poor countries, people report that poor health and associated expenses are among the top two or three causes of falling into poverty [44]. Thirdly, the past few years have seen an emerging literature on modelling disease-driven poverty traps that is based on explicit epidemiological and economic models. These models show, theoretically, how infectious disease could interact with economic drivers to create poverty traps [45,46]. In the next sections, we argue that zoonotic diseases are especially likely to act as poverty traps among poor livestock keepers in stressed ecosystems, both because of the high impacts of zoonotic diseases on both humans and livestock, and their generally unsatisfactory control. Even if the concept of poverty traps proves not to be widely applicable, the burdens of zoonotic and animal disease may be sufficient to contribute substantially to poverty among livestock keepers.

## 5. Zoonoses as important diseases among poor people

Zoonoses are diseases transmissible between animals (domestic and wildlife) and humans. Around 60% of all human diseases and around 75% of emerging infectious diseases are zoonotic [47,48]. The last decade has seen major progress in understanding the health burden of zoonoses and emerging diseases as the result of seven important studies (box 1). Poor livestock keepers are especially vulnerable to zoonoses due to their high contacts with livestock, their consumption of livestock products and their limited access to health provision, both for themselves and their animals.

Box 1. **Studies that advanced understanding of zoonoses.**

1. The first global assessment of emerging diseases inventoried all diseases emerging between 1930 and 2004 [49] and was updated in 2012 [50]. Overall, 76% of emerging diseases were zoonotic. While most emergence events were detected in developed countries, most high-burden emerging diseases affected developing countries to a greater extent. Moreover, the study found that in recent years, relatively more emerging diseases were detected in developing countries. Another study reviewed human infectious disease outbreaks from 1880 to 2013 [51]. In all, 65% of diseases identified were zoonoses, and these were responsible for 44% of outbreaks; while human-specific diseases exhibited a significant decline over this period, zoonoses and vector-borne disease exhibited significant increase.
2. The Global Burden of Disease first assessed disease in 1990 and there were important updates in 2006 and 2012 (http://www.who.int/healthinfo/global_burden_disease/gbd/en/). Unfortunately, zoonoses are not distinguished as a category, many important zoonoses (such as rabies) are omitted and, when diseases have zoonotic and anthroponotic components, these are not distinguished. However, literature estimates of zoonotic components suggest that 98.6% of the global burden of zoonotic disease is in poor countries and 1.4% in rich countries [43].
3. The first mapping of zoonoses and poverty found these were strongly correlated. Moreover, nearly all the human health burden of zoonotic disease in poor countries was due to endemic zoonoses: billions of illnesses and millions of deaths every year [50].
4. In 2015, the World Health Organisation released the first assessment of the burden of food-borne disease [52]. This study assessed 31 hazards for which there was sufficient evidence for global assessment. Together, these hazards caused at least 420 000 deaths and a burden of 33 million disability-adjusted life years (DALYs): comparable to malaria, HIV/AIDS or tuberculosis. Unsurprisingly, most of the burden (98%) fell on developing countries. Twenty of these hazards, responsible for 61% of the food-borne disease burden, were zoonoses.
5. A landmark study by the World Bank estimated the costs of major emerging zoonoses between 1990 and 2006. The study estimated that zoonotic outbreaks are currently costing the world $6.7 billion a year [53]. Furthermore, an investment of $1.9–$3.4 billion could reduce the probability of pandemics and other major outbreaks at a value of $37 billion a year. A cost–benefit analysis, which corrects for the very low probability of pandemics, shows that benefits far exceed costs in all plausible scenarios. In a related study, the World Bank and Taffs forum analysed animal health data for the years 2006 through 2009 as reported by member countries [54]. Half of the disease losses were due to zoonotic diseases and half to non-zoonotic diseases. Although extensive under-reporting of notifiable diseases in developing countries calls the quantitative estimates into question [50], the distribution of costs between zoonotic and non-zoonotic disease is plausible.

## 6. Escaping the poverty trap of endemic zoonoses

Endemic zoonoses are continually present to a greater or lesser degree in certain populations. Examples are cysticercosis, brucellosis, bovine tuberculosis, leptospirosis and food-borne zoonoses. These endemic zoonoses typically impose higher animal and human health burdens than outbreak zoonoses but are often a lower priority for governments and donors [43]. There is widespread consensus that most endemic zoonoses are better controlled in the animal host than the human victim and the historical record shows that where major endemic zoonoses have been controlled successfully, this has been the result of concentrating control efforts on the animal reservoir. Using this approach, brucellosis, tuberculosis, rabies, salmonellosis, cysticercosis, trichinellosis and others have been controlled successfully in many countries [55]. Economic assessments of these initiatives have shown that control of zoonoses is highly attractive [56]. One review reported a wide range of benefit-to-cost ratios, but all found that the benefits were higher than the costs. The median ratio of benefits to costs was around four to one with human health benefits at least equal to animal health benefits and often greater [57].

Yet despite the clear economic advantages of control in the animal host, most endemic zoonoses are not subject to active control in developing countries. By definition, zoonoses occur at the interface of human, animal and ecosystem health. This means the impact of zoonoses is at once both wider and less likely to be assessed and managed than diseases that fall comfortably within one sector: as a result, many zoonoses are considered neglected diseases.

In the DDDAC project, featured in this special edition, we conducted a rapid assessment of preventive and curative treatment costs for humans and animals in the study site in northern Kenya (box 2). We found there were no active campaigns for control of zoonotic diseases and that much of the responsibility and cost of both animal and human healthcare fell on households. Although we were not able to distinguish the proportion of spending attributable to endemic zoonoses, in general households spent very little on human preventive care, and somewhat more for preventive animal health, while they incurred substantially higher costs for curative human and animal treatments.

Box 2. **Health expenditures among pastoralist families in northeast Kenya.**

The DDDAC Kenyan case study focused on Rift Valley fever (RVF), a major emerging zoonosis. The case study took place in irrigated areas and adjacent pastoralist rangelands in order to investigate the relationships between land-use change, poverty and disease dynamics. During the study, it emerged that multiple zoonotic and non-zoonotic diseases were present and that these were frequently confused and misdiagnosed due to similar clinical presentations, so it was not possible to allocate expenses by disease. One substudy surveyed overall self-reported expenditure on human and animal health, using a rapid one-page assessment tool. (All research activities obtained relevant ethical clearance as described elsewhere.)

In total, 222 households were interviewed and asked about their expenditure for treatments the last three times someone in the family had been sick. In order to capture hidden costs from lost income, the number of days missed at work or in school was also captured, the latter because it is potentially poverty-promoting. Data were also collected on how much households spent annually on preventive measures, including mosquito nets, health insurances, boiling or other water treatments, vaccination and routine child health visits, deworming and vaccination of animals, treatments for flies or ticks, animal insurances and other health preventive measures for both humans and animals. As it is common never to get a diagnosis, people were asked for any disease.

The average household had 2.9 adults (range 1–10) and 4.6 children (range 1–11). The most commonly held livestock were small ruminants, followed by cattle (table 1). Costs per tropical livestock unit (TLU) varied greatly, with many households not treating their livestock at all. (Owing to the fact that few people kept poultry, and only 12 people had treated them, the cost for treatment per TLU makes this estimate very high; in terms of cost per animal treated, least was spent on poultry.)

**Table 1.** Last year expenditure on treatment of sick livestock by pastoralist households in Kenya.

	proportion households keeping (%)	average herd size (range)			costs for treatment last year	
		adult animals	young less than 1 year	TLU	KSH/animal	KSH/TLU
cattle	73.40	8.9 (0–68)	7.8 (0–70)	6.4 (0–49.5)	101.0 (0–500)	268 (0–1429)
sheep or goats	87.80	30.2 (0–309)	25.8 (0–224)	4.3 (0–39.5)	43.4 (0–300)	567 (0–4000)
poultry	27.50	3.3 (0–45)	^a^	0.02 (0–0.4)	5.4 (0–60)	538 (0–6000)
donkeys	10.40	0.2 (0–6)	0.1 (0–5)	0.2 (0–5.6)	148.1 (0–1000)	204 (0–1250)

^a^Not calculated for poultry due to tool only assessing less than 1 year as young.

None of the surveyed households invested in insurance for either family or animals. In terms of expenditure on preventive measures, the survey revealed that the highest level of expenditure was on mosquito nets, with households spending an average 120 KSH per year *per capita* (table 2).

**Table 2.** Last year expenditure on different preventive measures in pastoralist households in Kenya.

preventive costs per family	average annual cost (KSH)
mosquito nets/family member	120 (0–600)
water treatments/family member	1.4 (0–200)
child vaccination and routine checks/child	66 (0–833)
other preventive costs/family member	84 (0–2500)
total preventive cost/family member	245 (0–2800)
animal deworming/TLU	458 (0–10 000)
animal vaccination/TLU	235 (0–5714)
animal fly/tick treatments/TLU	239 (0–6000)
other preventive costs for livestock/TLU	329 (0–8000)
total preventive cost/TLU	1268 (0–29 000)

The survey asked households about the incidence of disease during the previous two weeks prior to survey administration. Survey results revealed an average of 2.2 disease incidents during this period, which ranged between zero and six. Based on the last three disease incidents at a household level, we found that the average direct costs spent on treatment were 306 KSH, but could be as high as 5300 KSH, with medicine usually constituting the bulk of these costs (table 3). The poverty-promoting aspect of disease is demonstrated by the fact that a family member could lose up to 10 days of work, or up to 7 days of school.

**Table 3.** Direct and indirect costs incurred as a result of human illness among pastoralist households in Kenya.

per disease occurrence, based on three most recently experienced diseases		
	mean (range)	proportion of total costs (range) (%)
medicine costs (KSH)	155 (0–2500)	47.6 (0–100)
travel costs (KSH)	83 (0–3000)	24.7 (0–100)
other costs (KSH)	68 (0–2000)	27.7 (0–100)
total costs (KSH)	306 (0–5300)	
days away from work	1.1 (0–10)	
days away from school	1.2 (0–7)

As families reported more than two incidents of disease in the family over the last two weeks, and an average cost of 306 KSH per disease incident, an average family could experience costs around 17 000 KSH per year, not including the indirect costs of lost incomes. Consequently, spending an average of 245 KSH per household member to prevent disease every year seems very little.

By contrast, the reported expenses for animal disease prevention were higher, at 1268 KSH per TLU, with costs of treatments being lower. It thus seems that people do tend to invest relatively more in prevention for animals to remain healthy compared with humans, potentially because they are considered an asset worth protecting.

Our findings align with the general conclusion that when poor communities are left to manage disease themselves, there is high willingness to pay for curative treatments but low willingness to pay for preventive action, even when this is likely to be much more effective and less costly. The case of human vaccinations for common diseases, which is generally considered a public good and vaccination an important objective, is illustrative. In most countries, there are free vaccination programmes for children and a research agenda on how vaccination rates can be increased by providing parents with incentives, such as money, goods or vouchers or requiring vaccination for school participation [58]. By contrast, vaccinations for most endemic zoonoses of animals are often not available or available only if paid for, and uptake is very low, outside of externally funded campaigns. Although most farmers tend to be willing to pay for curative veterinary services to some extent, a review of privatization processes in developing countries concludes that preventive veterinary medicine usually is considered a public good, and it may not be possible to privatize fully [59,60]. Our rapid assessment (box 2) indicates a relatively higher willingness to pay for animal prophylaxis than for human.

## 7. When official responses to disease deepen the poverty trap of zoonoses

In contrast with endemic zoonoses, outbreak zoonoses usually elicit responses from the public sector and donors. While disease control is an essential function of animal and human health systems, and the benefits of successful and cost-effective control are immense, unfortunately, control efforts, especially those targeting livestock owned by poor people and pastoralists are often limited in effectiveness. Worse still, attempts to control outbreaks can have unintended consequences that can be more serious than the outbreak itself. These include the direct loss of livestock, often not fully compensated for, but also indirect effects when consumers reduce consumption of animal source foods affecting the entire value chain. Unintended, and often unmeasured, consequences of disease control include the diversion of condemned food to human consumption and nutritional impacts from reduced animal source food consumption. For example, a 2006 avian influenza outbreak resulted in mass removal of chickens in Lower Egypt; this in turn probably led to an increase in childhood stunting as a result of reduced animal-source food intake [61].

Official and market-based responses to zoonoses are often magnified, given the dynamics of relationships and behaviour in the broader agri-food value chain. In many cases, the response—and burden—of disease is imposed at the production level, upon individual farmers with limited capacity and incentive to prevent and control the incursion of zoonoses. However, the actions and behaviour of other actors in the value chain may serve as risk factors for disease, but they are often not the focal point of public policy. For instance, distribution channels for livestock in many developing countries tend to be both uncoordinated and replete with market power (monopoly as sellers, monopsony as buyers) among intermediaries [62]. Where market power exists, prices are depressed for producers, reducing their incentives and ability to control disease. In addition, disease risk ‘hotspots’ are often concentrated among actors downstream, particularly traders and retailers who anonymously buy and sell animals from undifferentiated sources and can spread disease through their actions.

Conversely, the lack of attention given to downstream actors in the context of zoonoses reduces the ability of policymakers to leverage the support of these actors in the control of disease. In the context of the 2007 RVF outbreak, downstream actors such as traders, processors (particularly labourers in abattoirs), retailers and petty service providers (tea shops, scrap collectors, etc.) faced considerable economic losses from disease by virtue of the idling of production imposed by animal movement controls [63]. However, unlike farmers, such actors were not provided with any form of compensation. Similar stories can be found in the context of avian influenza [62]. At the same time, awareness campaigns centred on focal actors in the system can play an important role in the control efforts—Nigeria and Ghana were cited as examples of the effectiveness of such efforts in the case of avian influenza [62].

Given these dynamics, Rich & Perry [64] pointed out the need to consider the broader *system* in which livestock diseases and zoonoses take place as a means of better understanding and targeting disease control programmes. Even in the most rudimentary of production environment, livestock value chains can be complex, comprising a multitude of actors with different value systems, incentives and capacities to control disease. The lack of coordination among such actors in most developing country value chains makes aligning such incentives even more difficult. However, by understanding how the system works and identifying who the actors are, it facilitates a deeper understanding of the contextual drivers that shape and influence zoonoses, potentially making disease control efforts more effective.

As part of the DDDAC Kenya case study, featured in this special edition, we highlight the systems modelling used to better understand and communicate disease control (box 3). First, from a technical standpoint, systems models provide an excellent platform for directly overlaying socio-economic relationships with biological and epidemiological phenomena to highlight the feedbacks that exist between them and better address the consequences associated with disease. Most economic impact assessments of animal diseases and zoonoses highlight the ‘one-way’ effects associated with disease by translating the output of disease simulations into an economic model of some form [66]. However, feedback effects exist between the evolution and spread of disease and individual behaviour [67–69]. That is, disease outbreaks influence individual and collective decision-making at various levels within the value chain, in terms of inter alia production decisions (e.g. treatment options, breeding response, feed use), marketing decisions (e.g. distress sales to markets) and consumption decisions. In turn, these behaviours can (and will) influence the progression of disease in subsequent periods. By addressing these feedbacks within an impact assessment framework, one can better predict the impact of disease from economic and epidemiological perspectives and identify leverage points for intervention. For instance, improving awareness and incentives associated with distress sales would remove an important trigger point for the spread of disease.

Box 3. **A systems model for understanding RVF in Kenya.**

The systems model developed integrated the epidemiological spread of RVF through mosquitoes, livestock herd demographics and downstream marketing of livestock to meat markets. The epidemiological model consists of three components. First, it models the population dynamics of *Aedes* and *Culex* mosquito populations using a state-transition model of mosquito population classes, with population growth triggered by changes in rainfall that create reservoirs for mosquito spread. The mosquito growth model is then linked to a state-transition model of disease transmission (S-I-R) from mosquitoes to livestock.

The herd demographic model is based on the DynMod model of Lesnoff [65] that tracks the growth of cattle herds in pastoral settings. It distinguishes between cattle gender and age classes (calves, pre-adult and adult) and uses transition probabilities to calculate the movement of cattle from one age class to another, as well as for commercial off-takes and deaths. Death rates in this model distinguish between natural deaths and those attributed to RVF, where the latter come from the S-I-R model. An additional feedback exists between the herd demographic model and the disease model in that births from the herd model add to the pool of potential disease-susceptible cattle.

Past outbreaks of RVF resulted in the cumulative loss of thousands of human lives. The 2000 outbreak in Saudi Arabia led to the imposition of trade bans of live animals from the Horn of Africa (Ethiopia, Somalia and Kenya) that had devastating economic impacts: one study estimated that total economic value-added in the Somali region of Ethiopia fell by US$132 million because of these trade bans, a 42% reduction compared with normal years (A. Nin Pratt and others 2005, unpublished data). Rich & Wanyoike [63] estimated that RVF induced losses of over KSH 2.1 billion (US$32 million) to the Kenyan economy, based on its negative impacts on agriculture and other sectors (transport, services, etc.) alike.

A second benefit of using systems models for economic assessment is their ability to influence priority setting by stakeholders. Homer & Hirsch [70] reflected on the utility of system dynamics models in addressing broad public health issues related to the interactions of chronic diseases, their management and models of more effective service delivery; system dynamics modelling can generalize scenarios and decision rules associated with vaccination policy of eradicable infectious diseases such as polio [71]. Such models need not be ‘black boxes’ designed in the absence of stakeholder participation. Indeed, a systems modelling paradigm known as ‘group model building’ encourages the development of both qualitative and quantitative system dynamics models in conjunction with stakeholders directly [72–74], with recent advances taking spatial phenomena into account [75]. Such models have been used in both developed and developing country settings, and provide an opportunity to generate better models in difficult data-collection environments and which have greater stakeholder buy-in [76].

**Figure 1.**
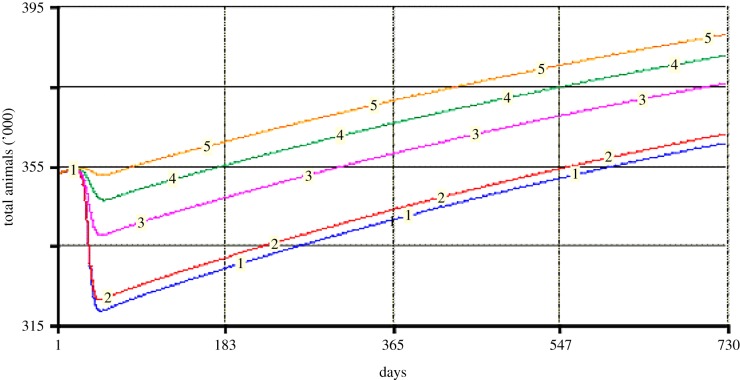
Potential effect of vaccination delay on cattle stock size. 1, no vaccination; 2, four weeks delay; 3, two weeks delay; 4, one week delay; 5, no delay.

In the DDDAC project, a system dynamics model was used to help decision makers understand how the effects of disease in terms of animal losses could be mitigated through timely vaccination of animals. Mounting responses to disease outbreaks in remote pastoralist areas is often difficult, and there is a tendency for decision-makers to accept delay as inevitable. However, the model allowed them to explore the relation between timeliness and losses of animals represented in terms of potential stock sizes decline, which the model predicts through its demographic module. Notably, losses associated with a four weeks delay are almost the same as the losses incurred when no vaccination is done (figure 1). They can motivate more investment in preparedness and a timely response. On the other hand, the model can help avoid the costs which epidemic outbreak control imposes on the public sector and pastoralists, by discouraging a vaccination response when it is too late to make a difference.

## 8. Conclusion

Development initiatives to address poverty have been largely supply-driven, often following a missionary model whereby outsiders bring their top-down solutions to communities in need. Understanding the complex interactions between agro-ecosystems, culture, values, institutions, behaviour and possibly even genetics may give better insight into whether solutions succeed or fail, or prove appropriate or not, as well as aid in developing new approaches to poverty reduction. Disease arises from interactions between hosts, vectors, environments and pathogens and recent studies have confirmed the large burden of endemic zoonoses and the effectiveness and high cost-effectiveness of control in the animal reservoir. Despite this, our case study illustrates how communities bear heavy burdens of human and animal disease and devote their limited resources to therapeutic rather than preventive measures. In contrast with endemic zoonoses, widely perceived as neglected, outbreak zoonoses elicit strong responses from the public, national governments and donors. These findings support the hypothesis that both the high burdens of endemic disease and the unanticipated effects of disease control may act as poverty traps. More systematic approaches to understanding downstream effects of disease (including on markets and nutrition) can lead to better responses.
